# Short- and Long-Term Efficacy of Modafinil at Improving Quality of Life in Stroke Survivors: A *Post Hoc* Sub Study of the Modafinil in Debilitating Fatigue After Stroke Trial

**DOI:** 10.3389/fneur.2018.00269

**Published:** 2018-04-25

**Authors:** Thomas P. Lillicrap, Christopher R. Levi, Elizabeth Holliday, Mark William Parsons, Andrew Bivard

**Affiliations:** ^1^Neurology Department, John Hunter Hospital, Newcastle, NSW, Australia; ^2^Hunter Medical Research Institute, Newcastle, NSW, Australia

**Keywords:** stroke, stroke recovery, fatigue, modafinil, quality of life

## Abstract

**Background:**

The phase-II modafinil in debilitating fatigue after stroke trial demonstrated that modafinil improves fatigue and quality of life in severely fatigued stroke survivors. For this study, we sought to examine the interaction between fatigue and quality of life after stroke and determine whether reducing fatigue resulted in improved quality of life. In addition, we followed up a subset of patients 12-months after the trial to assess the long-term outcomes of modafinil therapy.

**Methods:**

We used linear regression to analyze interaction between baseline fatigue, as measured by the multidimensional fatigue inventory (MFI), and quality of life, as measured by the stroke-specific quality of life scale (SSQoL); and between changes in MFI and SSQoL during treatment. Patients also took part in semi-structured interviews and study assessments 12-months after trial completion to assess long-term patterns of modafinil use, safety and efficacy.

**Results:**

MFI and SSQoL were significantly correlated at baseline (β = −1.975 95% CI −3.082, −0.869, *p* < 0.001), as were changes in MFI and SSQoL during treatment (β = −1.054 95% CI −1.556, −0.553, *p* < 0.001). 18 patients agreed to 12-month follow-up, of whom 5 had continued to use modafinil. Patients taking modafinil daily demonstrated sustained improvement of 33–38 points in MFI compared to baseline. Two adverse events were reported and there was no evidence of drug tolerance.

**Conclusion:**

Modafinil appears to be safe and, for at least some patients, effective long-term in fatigued stroke survivors. Alleviating fatigue has a significant relationship with improved quality of life.

**Clinical trial registration:**

https://www.anzctr.org.au/Trial/Registration/TrialReview.aspx?id=368268, unique-identifier: ACTRN12615000350527.

## Introduction

Approximately 40% of stroke survivors experience debilitating fatigue 3 months or more after their stroke which can negatively impact their quality of life, interfering with their ability to participate in rehabilitation and return to everyday activities such as work and socialization (1). We recently investigated the effectiveness of the wakefulness-promoting agent modafinil in stroke survivors as part of the modafinil in debilitating fatigue after stroke (MIDAS) trial (2). During MIDAS, participants reported a significant decrease in fatigue following 6 weeks of modafinil therapy (200 mg/day) and a concurrent improvement in self-reported quality of life. Following from the success of the MIDAS trial, we sought to identify if there was a significant relationship between the resolution of fatigue and improved quality of life during the trial and follow-up patients 12 months after the trial to identify if the reported improved quality of life was sustained 12 months after completion of trial participation. Although modafinil is not approved for stroke-related fatigue in Australia, it can be prescribed off-label by a neurologist or general practitioner and some patients had made use of this fact to continue taking modafinil at their own expense.

## Methods

### Study Population

Inclusion criteria for MIDAS were patients >18 years old with a history of stroke at least 3 months previously and a total score of 60 or higher across all domains of the multidimensional fatigue inventory (MFI) which has a healthy population mean score of 35–40 (3). Exclusion criteria were known contraindications to modafinil or an alternate diagnosis known to cause fatigue. During the MIDAS trial, each participant was randomized to either 200 mg/day of modafinil or placebo followed by a 1-week washout period and treatment crossover. This study was approved by the Hunter-New-England Area Health District Human Research Ethics committee. The long-term follow-up was approved as a sub study of the original trial. All participants provided informed written consent to both the original trial and, where applicable, the long-term follow-up sub study.

### Patient Assessments

Patients were assessed at baseline and following completion of each study arm (active and placebo as part of the crossover design) using the MFI, stroke-specific quality of life scale (SSQoL), and the Depression Anxiety and Stress Scale (DASS). The MFI assesses fatigue across 5 domains (general, mental, physical, reduced activity, and reduced motivation), where each domain is scored out of 20, the total is scored out of 100 and higher scores indicate more severe fatigue.

As part of the follow-up for the trial, participants were contacted by mail inviting them to take part in the sub study examining long-term patient outcomes. Those who consented underwent a semi-structured interview to determine whether and how they had continued taking modafinil, and their individual reasons for this decision. The patients were assessed for fatigue using the MFI and quality of life using the SSQoL, and their hospital records were audited for any new admissions or consultations related to adverse effects of modafinil.

### Statistical Analysis

Relationships between baseline MFI and baseline SSQoL scores and outcomes were assessed using linear regression. Beta coefficients with their 95% confidence intervals and Pearson’s correlation coefficients were estimated. Differences in pre- and post-treatment quality of life were compared to differences in pre- and post-treatment fatigue, which had been demonstrated to be improved by modafinil (2). Treatment effects and interactions between treatment and baseline measures were assessed using linear regression including fixed effects for participant, treatment (modafinil or placebo), baseline outcome measure, and interaction between treatment and baseline measure. For this analysis, treatment effect was measured as the within-patient improvement in MFI and SSQoL on modafinil vs placebo. However, it should be noted that SSQoL was a secondary outcome measure and MIDAS was not designed to specifically test this effect.

For the assessment of the long-term effect of modafinil on individual participants, the MFI and SSQoL scores of each patient were compared to their baseline scores and to their scores at the end of their 6-week modafinil treatment. All statistical analysis was programmed using SAS version 9.4 or SPSS version 23.0.

## Results

**Figure 1:**
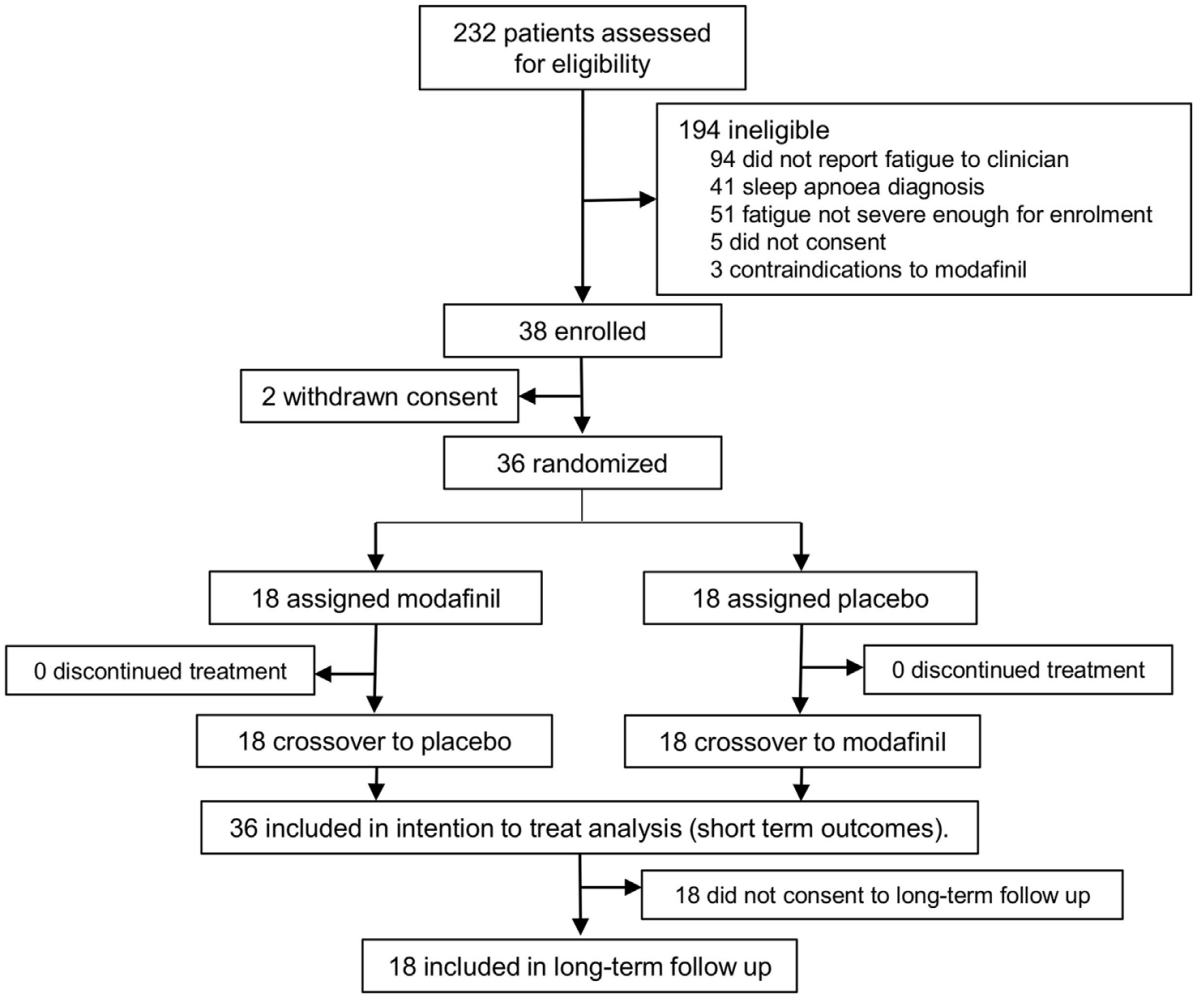
Patient flow for the modafinil in debilitating fatigue after stroke trial and subsequent long-term sub study. Patients were screened for severe fatigue and then randomized to either modafinil or placebo for 6 weeks, followed by treatment crossover after a 1-week washout period. Patients were contacted by mail 12 months after the conclusion of the trial and invited to take part in the long-term sub study. Those who consented to this sub study were interviewed and assessed for long-term outcomes.

36 patients completed the MIDAS trial (see Figure 1; 22 males, mean age 63, SD 15), at mean time of 9 months poststroke (range 3–38 months). Baseline MFI was 72 (SD 8.7) and mean SSQoL at baseline was 152.6 (SD 35.7). Modafinil was found to reduce total MFI by an average of 17.38 (95% CI 12.99, 21.76, *p* < 0.0001) and to improve SSQoL by an average of 11.81 (95% CI 2.31, 21.31, *p* = 0.0148) compared to placebo.

### Short Term

Baseline fatigue and quality of life were significantly negatively correlated, with each 1-point increase in the baseline MFI associated with a 1.975-point reduction in baseline SSQoL (see Table 1). The change in the total SSQoL score was associated with the change in MFI during modafinil treatment periods, with each 1-point decrease in the MFI corresponding to an improvement of approximately 1-point in the SSQoL (Table 1).

**Table 1:** Interactions between baseline scores and treatment effects for the 6-week treatment period tested in the original modafinil in debilitating fatigue after stroke trial.

Interaction	β	95% CI of β	*p*(<)
Baseline multidimensional fatigue inventory (MFI) vs baseline stroke-specific quality of life scale (SSQoL)	−1.975	−3.082, −0.869	0.001

Change in MFI vs change in SSQoL during treatment	−1.054	−1.556, −0.553	0.001

Baseline MFI vs change in MFI during treatment	−0.701	−1.0746, −0.3319	0.001

Baseline anxiety [Depression Anxiety and Stress Scale (DASS)] vs change in MFI during treatment	0.647	0.0875, 1.2080	0.023

Baseline anxiety (DASS) vs change in SSQoL during treatment	−1.2044	−2.402, −0.005	0.048

A greater reduction in MFI after modafinil therapy was associated with higher baseline MFI scores. Change in SSQoL during modafinil therapy was not predicted by baseline MFI (*p* = 0.2434). A higher level of baseline anxiety, as measured by the anxiety domain of the DASS, was associated with a smaller change in both the MFI and the SSQoL in response to modafinil therapy (Table 1). Baseline scores in other domains of the MFI or the DASS were not found to have significant interactions with treatment effect (all nominal *p*-values > 0.05).

### 12-Month Follow-Up

18 patients agreed to take part in the follow-up assessments. Of these, 13 patients were no longer taking modafinil, 2 were taking modafinil occasionally (1–3 times per week), and 3 had continued taking modafinil daily. Of the patients who were no longer taking modafinil, one reported that this was primarily due to the cost, two had experienced worsening anxiety while on modafinil and had discontinued the drug as a result, one reported that her fatigue had improved and therefore did not require the drug any longer, and nine reported no desire to continue treatment as the perceived benefits were not significant enough to continue. Both of the patients who took modafinil occasionally reported that the primary reason they did not use the drug regularly was the cost.

Patients who were not taking modafinil reported an average change in MFI from baseline of −8 points (median −6 points, range −23 to 6) and a change of 3 points from post-modafinil treatment score (median 4 points, range −17 to 18). The patients taking modafinil occasionally reported an average change in MFI from baseline of −15 points (median −15 points, range −11 to −19) and a change of 23 points from post-modafinil treatment (median 23 points, range 19–27). Finally, patients who had continued to use modafinil daily reported an average change in MFI from baseline of −36 points (median −36 points, range −33 to −38) and a change from post-modafinil treatment of −3 points (median 0 points, range −9 to 1).

The only adverse events or side effects reported were two cases of worsening anxiety reported by patients who had discontinued modafinil treatment as a result.

## Discussion

The fact that fatigue was significantly correlated with quality of life at baseline, as well as after treatment, suggests that the improvement in quality of life experienced by patients during the MIDAS trial was, at least in part, a direct result of alleviating fatigue. This *post hoc* sub group analysis of the MIDAS trial has observed that for every point improvement in fatigue on the MFI scale, participants quality of life improved by one point on the SSQoL. The results suggest that alleviating fatigue in stroke survivors can also be expected to improve quality of life. Previous studies have demonstrated that fatigue negatively affects quality of life in stroke survivors (4) but it has not been demonstrated that alleviating fatigue can improve quality of life poststroke.

The minimum clinically important change for the SSQoL (i.e., the magnitude of the change necessary to be meaningful for an individual patient) has been shown to be 10 points (5). Given the coefficient found in this analysis (approximately 1 point improvement in SSQoL for every 1 point improvement in MFI), any intervention aimed at improving quality of life through the resolution of fatigue would require a minimum reduction of 10 points in the MFI to achieve this threshold in the SSQoL. Patients in MIDAS experienced an average change of 17 points in the MFI with modafinil therapy, supporting the use of modafinil to meaningfully improve quality of life.

Higher scores in both MFI and the motivation domain of the MFI were associated with a greater treatment effect. The reduced motivation score makes up part of the MFI, therefore the treatment interaction of the motivation score cannot be separated from that detected for the MFI. These findings suggest that modafinil is particularly effective in patients who have apathy and resultant low motivation to participate in activities of daily living.

Modafinil continued to be well tolerated over an extended time frame in our elderly cohort who has recently had a stroke; the only reported adverse effect (anxiety) was a previously documented side effect of modafinil, which resolved after the drug was discontinued. It is also notable that patients continuing to take modafinil daily did not show signs of habituation or tolerance, with each of these patients demonstrating sustained benefit and MFI scores below the population mean (35–40) without increasing their dose of the drug.

Long-term modafinil use appears to offer ongoing benefits in terms of both fatigue and quality of life in some stroke survivors, with daily use required to obtain the greatest benefit. However, in Australia, many patients would require financial support to use modafinil daily. Patients taking modafinil long term will require continued monitoring for side effects, particularly those patients with higher levels of anxiety. The conclusions described here are limited by the small sample-size, and a self-selection bias caused by the fact that only those patients who perceived the most benefit from modafinil during the original trial chose to continue the drug long term.

In conclusion, within the limits imposed by our small patient cohort, we have shown that in severely fatigued stroke survivors modafinil is a safe and effective therapeutic option to improve fatigue and improve quality of life.

## Ethics Statement

This study was approved by the Hunter-New-England Area Health District Human Research Ethics committee. The long-term follow-up was approved as a sub study of the original trial. All participants provided informed written consent to both the original trial and, where applicable, the long-term follow-up sub study.

## Author Contributions

TL, AB, and CL wrote and proofed the manuscript. AB, CL, and MP designed the trial. TL collected the data. EH conducted statistical analysis.

## Conflict of Interest Statement

The authors declare that the research was conducted in the absence of any commercial or financial relationships that could be construed as a potential conflict of interest. The reviewer MR and handling Editor declared their shared affiliation.
